# Atomic mapping of Ruddlesden-Popper faults in transparent conducting BaSnO_3_-based thin films

**DOI:** 10.1038/srep16097

**Published:** 2015-11-03

**Authors:** W. Y. Wang, Y. L. Tang, Y. L. Zhu, J. Suriyaprakash, Y. B. Xu, Y. Liu, B. Gao, S-W. Cheong, X. L. Ma

**Affiliations:** 1 Shenyang National Laboratory for Materials Science, Institute of Metal Research, Chinese Academy of Sciences, Wenhua Road 72, Shenyang 110016, China; 2 Rutgers Center for Emergent Materials and Department of Physics & Astronomy, Rutgers University, Piscataway, New Jersey 08854, USA

## Abstract

Doped BaSnO_3_ has arisen many interests recently as one of the promising transparent conducting oxides for future applications. Understanding the microstructural characteristics are crucial for the exploration of relevant devices. In this paper, we investigated the microstructural features of 0.001% La doped BaSnO_3_ thin film using both conventional and aberration corrected transmission electron microscopes. Contrast analysis shows high densities of Ruddlesden-Popper faults in the film, which are on {100} planes with translational displacements of 1/2a < 111 > . Atomic EELS element mappings reveal that the Ruddlesden-Popper faults are Ba-O layer terminated, and two kinds of kink structures at the Ruddlesden-Popper faults with different element distributions are also demonstrated. Quantitative analysis on lattice distortions of the Ruddlesden-Popper faults illustrates that the local lattice spacing poses a huge increment of 36%, indicating that large strains exist around the Ruddlesden-Popper faults in the film.

Owing to the high electrical conductivity and optical transparent properties, transparent conducting oxides (TCOs) have received extensive attentions and demands for a large number of applications, such as optical electrode in solar cells, flat-panel displays, and sensors etc^1^,^2^,^3^. Much work on TCOs has been mainly focusing on binary oxides including In_2_O_3_, SnO_2_ and ZnO in the past few years, which are either expensive due to the rareness of indium, or inadequate transitivity of ultraviolet light and thus reduce the efficiency of light conversion^4^,^5^. Recently, doped perovskite BaSnO_3_ (BSO) has arisen many interests due to the large band gap and high carrier mobility^6^,^7^,^8^. In the single crystal, the carrier mobility can be attained as high as ~320 cm^2^ V^−1^ S^−1^ (ref. 8) comparable to the value of 100 ~ 160 cm^2^ V^−1^ S^−1^ previously reported in standard TCOs^9^,^10^, enabling great potentials for BSO to be used in practical applications.

Generally, in contrast with the bulk, thin films are not only the existing status for many materials in devices, but they also promise many novel properties due to the unique structural features^11^,^12^,^13^,^14^,^15^. For example, misfit dislocations (MDs) are commonly observed in thin films while one dimensional electron chains are found at these MDs in magnetic thin films^11^. And interfaces, such as film/substrate interfaces, twins or stacking faults, can provide another degree of freedom to mediate the property of thin films, where superconductivity^13^, ferroelectricity^14^, and novel magnetic phase^15^ can be generated. At these interfaces, both the ionic potential and electronic state are distinct from those of the bulk matrix and account for the superior properties^12^. As a result, atomic mapping of the structural features in thin films are then crucial for understanding the various properties of the thin films. For the structures of BSO based thin films, previous studies have mainly focused on dislocations^16^,^17^. Mun *et al.* studied the relations between the density of dislocations with film growth temperatures^16^. Wadekar *et al.* demonstrated the influence of lattice mismatch on dislocation densities of films^17^. However, the detailed structural information especially various interfaces of the BSO thin films are still inadequate.

In this work, utilizing the conventional and advanced aberration-corrected transmission electron microscopy techniques, we characterized the detailed microstructures of La-doped BSO thin film and the atomic configurations of the corresponding planar defects. High densities of Ruddlesden-Popper (RP) faults are found on {100} planes with translation displacements of 1/2a < 111 > . The element distributions and lattice distortions around the RP faults are also explored.

## Results

### Microstructures of BSO thin film

**Figure 1:**
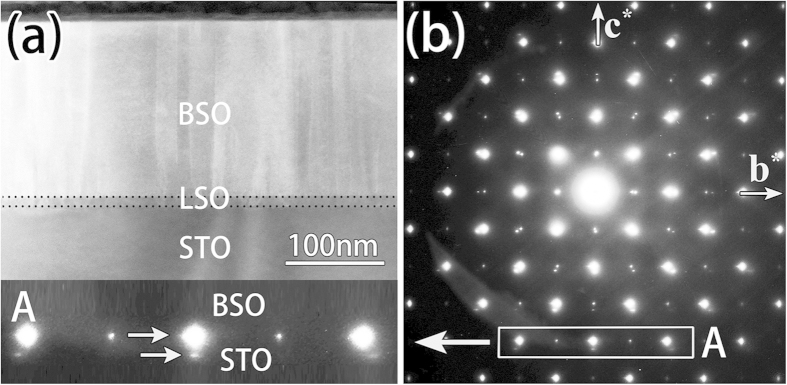
(**a**) A cross-sectional low magnification HAADF-STEM image of BSO film on LSO-buffered STO (001). The interfaces are marked by dotted lines. (**b**) A selected area electron diffraction pattern including both the film and the substrate in [100] direction. The elongation of BSO spot parallel to the interface is indicated by the rectangular frame “A” and its enlargement at the bottom of the left column.

The bulk BSO has an ideal cubic perovskite structure (*a* = 0.4116 nm), and is a transparent semiconductor with band gap more than 3.1 eV (ref. 18). After doping a little amount of La, it exhibits a large electron conductivity with a small lattice expansion^6^,^7^. Figure 1a is a low magnification HAADF-STEM image of BSO thin film. The BSO is buffered with a LSO layer to reduce the lattice mismatch between the film and the substrate (STO). The interfaces of BSO/LSO and LSO/STO are indicated by dotted lines (more details are shown in Figure S1 in Supplementary Matreial). Figure 1b is a selected area diffraction pattern containing the film and the substrate in [100] zone axis. The composite diffraction pattern mainly arises from the diffraction of the BSO film and the STO substrate, since the LSO buffer layer is very thin and its contribution to the diffraction pattern is negligible. The lattice parameter of BSO derived from the diffraction pattern is about 0.412 nm, close to the value of the bulk, signifying that the mismatch between the film and the substrate is released. Generally, lattice mismatch is released by generating MDs at or near the interfaces^19^,^20^,^21^,^22^, and the formation of the MDs is often related to evolution of threading dislocations (TDs)^23^. Thus, it is expected that there are a large number of MDs and TDs in the BSO/LSO/STO thin film due to the misfit strain relaxation. In addition, the diffraction spots of BSO marked with a rectangular frame “A” in Fig. 1b are spike-like, which imply the presence of high-density planar defects in the film with the boundaries perpendicular to the film/substrate interface^24^,^25^. The distribution of the MDs, TDs and the existence of planar defects are revealed using diffraction contrast analysis below.

**Figure 2:**
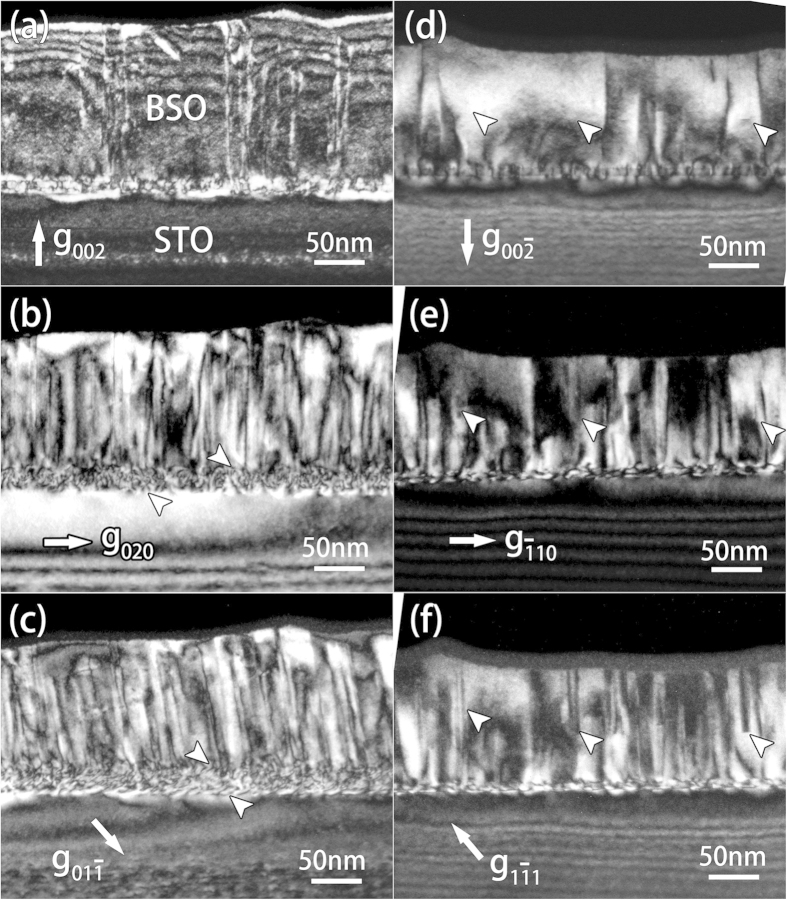
(**a**–**c**) Two-beam dark-field images of cross-sectional BSO/LSO/STO thin film by using (**a**) (002), (**b**) (020) and (**c**) (01–1) reflections of [100] pole. Contrasts of short dark lins near the interfaces (BSO/LSO and LSO/STO) are labeled by arrows in (**b,c**). (**d**–**f**) Two-beam dark-field images using (**d**) (00–2), (**e**) (−110) and (**f**) (1–11) reflections of [110] pole. Arrows in d-f reveal contrasts of planar defects under different reflections.

**Figure 3:**
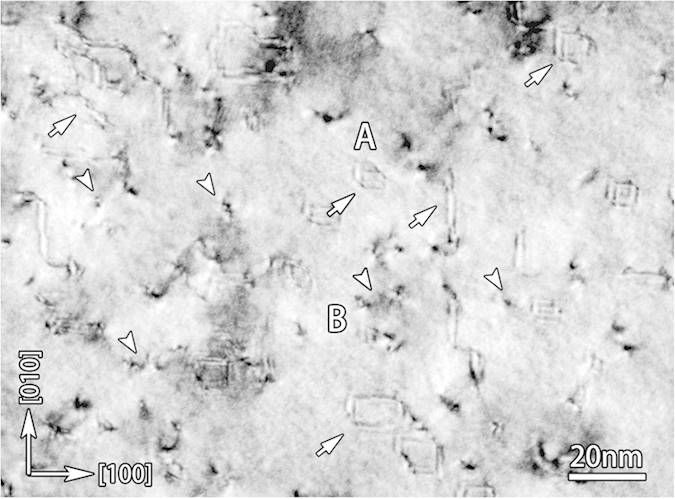
A bright-field image of a plan-view sample only containing BSO film near [001] direction. High density of SFs (line contrasts A) and threading dislocations (spot contrasts B) in the film are clearly seen, and labeled by two types of arrows respectively.

Figure 2a–c are two-beam dark-field images of a cross-sectional sample using different reflections of [100] pole, whereas Fig. 2d,f are those of a cross-sectional sample using [110] pole. The diffraction contrast can be analysed in two folds. First, contrasts of short dark lines (indicated by arrows in Fig. 2b,c) lying on the locations of interfaces (BSO/LSO and LSO/STO) are observed, which result from the MDs (more details are shown in Figrue S3 and S4 in Supplementary Material). They are nearly out of contrast using out of plane reflections, e.g. (002) in Fig. 2a. According to the dislocation contrast extincton criterion: **g·b** **=** **0**, the Burgers vectors of these MDs should be parallel to the interface. When we tilt the sample far away from the [100] pole in Fig. 2c, the contrasts of MDs are only visible in one direction (marked by arrows) which is consistent with the character of edge dislocations with the Burges vectors of a < 100 >^19^ and is also confirmed by Figure S4 in Supplementray Material. These high density MDs often connect with TDs (Fig. 2b,c), most of which are out of contrast using out of plane reflections as that in Fig. 2a. These TDs are also a < 100 > type dislocations. Second, the contrasts marked by arrows in Fig. 2d,f are only visible when (1–11) reflection (Fig. 2f) is selected to image; while such contrasts are very weak and sometimes hardly observed when other diffractions (Fig. 2e,d) are used. Obviously, these contrasts should result from planar defects of stacking faults (SFs). Due to the strong contrasts of dislocations, the distribution of these SFs can be hardly revealed in the cross-sectional samples. Thus, plan-view investigation is performed to demonstrate the density and the distribution of the stacking faults. Figure 3 shows a bright-field image of a plan-view sample only containing the BSO film. High density SFs (A) and TDs (B) are identified, which are indicated with the arrows of variant fashions. All the SFs are on {100} planes and some of them intersect with TDs. These characters of the SFs should be strongly connected with their detailed structural configuration and are further demonstrated below.

### Identification of RP faults and SFs of 1/2a < 110 > in the film

**Figure 4:**
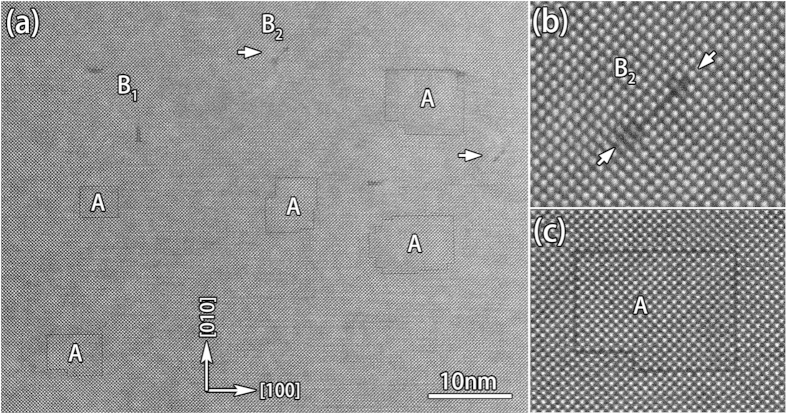
(**a**) A low magnification of HAADF-STEM image of a plan-view sample only containing BSO film in [001] direction. Polygon-like shapes comprised by the SFs are labeled by A. B_1_ denotes a < 100 > type perfect threading dislocations. B_2_ denotes extended threading dislocations with a short SF on (−110) plane terminated by two 1/2a[−110] partial dislocations. (**b**) An atomic resolved HAADF-STEM image of B_2_. The arrows indicate two excess half layers of the 1/2a[−110] partial dislocations. (**c**) An atomic resolved HAADF-STEM image of the SF (RP fault) at left bottom in (**a**). Lattice distortion around the SF is clearly seen.

Figure 4a is a low magnification HAADF-STEM image of a plan-view sample only containing the BSO film in [001] direction. The SFs labeled by A indeed lie precisely on {100} planes and they appear in regular shapes. One of them is enlarged and shown in Fig. 4c. The projection of translational displacement along (001) plane can be deduced to be 1/2a < 110 > . In perovskite materials, there are normally two kinds of translational displacements involved with the stacking faults: 1/2a < 111 >^25^,^26^ and 1/2a < 110 >^27^. In the former case, the projected translational displacements along < 100 > direction are always 1/2a < 110 > , while in the latter case it should contain projected translational displacements of 1/2a < 100 > along one of the < 100 > directions. Since all the SFs that we have observed in both plan-view and cross-sectional samples are with projected translational displacements of 1/2a < 110 > , the translational displacements of the SFs here in the film should be 1/2a < 111 > . And such kind of SFs are all on {100} planes, they are normally named in terms of Ruddlesden-Popper (RP) faults^26^,^28^.

For the TDs in Fig. 4a, two types are identified: one is perfect dislocation of a < 100 > (B_1_) and the other is extended dislocation of a < 110 > (B_2_). The extended dislocation includes two 1/2a < 110 > type partial dislocations (marked by an arrow in Fig. 4b) connected with a short SF. The short SF lies on {110} planes and has a translational displacement of 1/2a < 110 > , which is the same as the partial dislocation. The formation of such SFs is proposed to be associated with the climbing of a 1/2a < 110 > partial TD, for example, the partial TD of 1/2a[−110] in Fig. 4b shows a climbing along the [110] direction.

### Atomic mapping of the element distributions at the RP fault

**Figure 5:**
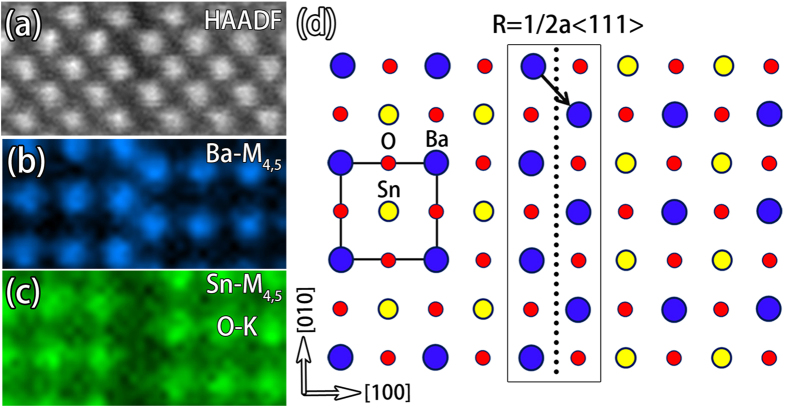
(**a**) An atomic resolved HAADF-STEM image of the RP fault. (**b**) The EELS element map of Ba by using Ba-M_4,5_. (**c**) The EELS element map of both Sn and oxygen by using Sn-M_4,5_ and O-K, respectively. (**d**) An atomic model of the RP fault. Ba, Sn, O are denoted by blue, yellow, and red spheres, respectively. The unit cell is outlined by the black square frame.

In complex materials, SFs can be classified into two types: conservative SF and non-conservative SF. This classification depends on the relation between the lattice periodicity along normal direction of the SF and the magnitude of the translational displacement^27^. For the conservative SF, the stoichiometric ratio is consistent with the perfect area, while for a non-conservative, element excess can be found. The element information of these two kinds of SFs can be elucidated by element mapping. For the RP fault, as the displacement vector is not an integral multiple of the periodic vector of {100} planes, which is a < 100 > , element excess can be expected. Figure 5 shows the atomic EELS element mappings of the RP fault. Ba distribution is imaged by using Ba-M_4,5_ as shown in Fig. 5b. As the M_4,5_ of Sn features a delayed profile directly extending to the K edge of oxygen (Supplementary Material), we thus use both of these edges to show the distribution of Sn. Figure 5c shows the element mapping result at two sides of the RP fault using Sn-M_4,5_ and O-K. These results demonstrate that the distribution of Sn can be indeed revealed even in the presence of part of oxygen K edge. According to the distributions of both Ba and Sn, it is feasible to construct the structural model of the RP fault in Fig. 5d. Such kind of structure contains a half unit cell of NaCl type BaO at the interface and breaks the Sn-O-Sn bond chains in the direction perpendicular to the interface, which is depicted by the black frame in Fig. 5d.

**Figure 6:**
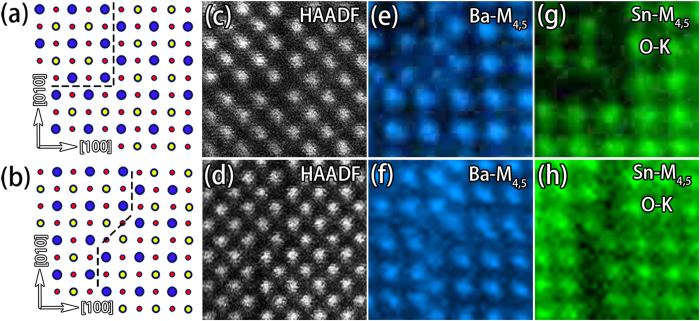
(**a,b**) Atomic models of two kinds of kinks at the RP faults. (**c–h**) HAADF-STEM images and corresponding Ba-M_4,5_ and Sn-M_4,5_ and O-K maps around the two kinks. Ba, Sn, O are denoted by blue, yellow, and red spheres, respectively.

In addition, considering the regular shapes comprised by these RP faults as shown in Figs 3 and 4a, kinks or steps at the corners of these regular shapes are expected to be prevailing in the film. The element distributions at these kinks reveal interesting behaviors. According to the EELS element mapping results, the kinks include two types: one consists of two {100} planes (Fig. 6a), the other contains one {110} and two {100} planes (Fig. 6b). Figure 6c–h show the HAADF-STEM images and element mapping of these two kinds. The kink in Fig. 6a can be understood by considering one RP fault on (100) plane intersecting with another RP fault on (010) plane, and of course is out of stoichiometry with an excess of Ba. For the kink in Fig. 6b, it comprises two RP faults on (100) planes and one short SF on (−110) plane. Although the two RP faults are out of stoichiometry, the short SF is stoichiometric. From the structural consideration, the perovskite BSO is composed of one layer of Ba-Sn followed by one layer of O-O along < 110 > directions, and the periodicity in these directions then should be 1/2a < 110 > . The translational displacement (1/2a < 111 > ) of the kink strtucture in Fig. 6b would not break the periodicity of BSO in < 110 > direction, thus the short SF on (−110) plane at the kink in Fig. 6b is stoichiometric.

**Figure 7:**
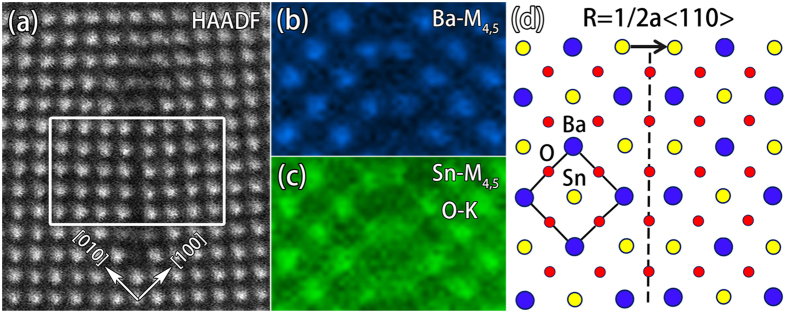
(**a**) An atomic resolved HAADF-STEM image of an extended dislocation of a < 110 > type. Part of the SF used to analyze is marked by the white square frame. (**b**) The EELS element maps of Ba by using Ba-M_4,5_. (**c**) The EELS element maps of both Sn and oxygen by using Sn-M_4,5_ and O-K, respectively. (**d**) An atomic model of SF with translational displacement of 1/2a < 110 > . Ba, Sn, O are denoted by blue, yellow, and red spheres, respectively. The unit cell is marked by the black square frame. The termination layer of SF is marked by the black dotted line.

In contrast to the RP fault, the SFs of 1/2 < 110 > on {110} planes reveal different element distributions. Figure 7a is a HAADF-STEM image of an extended dislocation of a < 110 > type where part of the SF between two partial dislocations of 1/2a < 110 > is marked by a white square frame and utilized for element analysis. Figure 7b shows the Ba maps by using Ba-M_4,5_. The segregation of Ba can be directly identified. Figure 7c is a map of both Sn and O by using Sn-M_4,5_ and O-K. Based on these element information, the possible atomic model of the corresponding SF is proposed in Fig. 7d and O-O termination at the SF is deduced. Althought the segregation of Sn and Ba at the SF in Fig. 7b,c is obvious, the overall stoichiometric ratio remains the same with the perfect area, as the periodicity is maintained in [1–10] direction.

### Lattice distortions and strains at the RP fault

**Figure 8:**
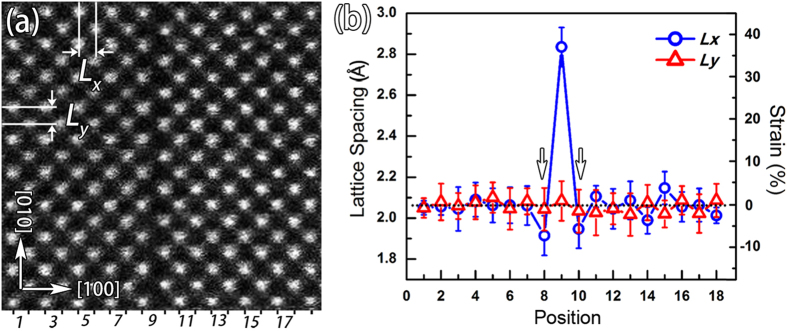
(**a**) An atomic resolved HAADF-STEM image of the RP fault used to quantitatively analyze the lattice spacing variations. *Lx* denotes the local horizontal lattice spacing between two nearby atoms, while *Ly* denotes the local vertical lattice spacing between two nearby atoms. Statistic lattice spacing across the RP fault is obtained by averaging *Lx* and *Ly* between two columns parallel to the RP fault. The positions of every two columns are indicated by the numbers. (**b**) The line profile of the statistic lattice spacing (*Lx* and *Ly*) across the RP fault. *Lx* shows an obvious increase at the SF, whereas a little decrease marked by arrows at two sides of the RP fault. *Ly* shows unnoticeable variations across the RP fault.

Despite the translational displacement and element distribution, lattice distortions as well as associated strains are also important characters in relation to the RP faults. As the RP faults in BSO thin film are on {100} planes with translational displacements of 1/2a < 111 > , we quantitatively analyzed the lattice distortion and the corresponding strain in terms of lattice spacing around the interface. Figure 8a is an atomic resolved HAADF-STEM image of the RP fault on which the revelent analysis is based. To clearly demonstrate the lattice distortions, positions of each atom column are determined based on two dimensional Gaussian fitting of the intensity profile of each atom column^29^. And lattice spacing *Lx* and *Ly* are defined. *Lx* is the horizontal distance between two nearby atoms representing the local horizontal lattice spacing, while *Ly* is the vertical distance between two nearby atoms indicating the local vertical lattice spacing. As the SF is on (100) plane, lattice distortion parallel to the (100) plane is similar. Thus *Lx* and *Ly* parallel to the (100) plane of the RP fault are averaged to reduce noise from HAADF image. Figure 8b is the line profile of the averaged value of *Lx* and *Ly* and corresponding strain across the RP fault. The position number denotes the location of *Lx* and *Ly* used to average (also seen in Fig. 8a). Far away the RP fault, *Lx* and *Ly* are normalized as the bulk value of 0.206nm. Obvious lattice distortions are found for the local lattice spacing *Lx* near the RP fault. At the RP fault (position number 9), *Lx* has an extraordinary increment (~0.283 ± 0.01 nm) leading to a nominal tension strain as high as 36%. At the two sides of the RP fault (position number 8 and 10), *Lx* shows a little diminishment with a compression strain of about −6% ~ −7%. Whereas, the local lattice spacing *Ly* remains relatively constant across the RP fault, indicating the lattice distortion mainly existing on the normal direction of the RP fault. The complex behavior of *Lx* is related to the unique atom arrangement of the RP fault. As mentioned above, such kind of SF in fact comprises a half unit cell of NaCl type BaO, which has a lattice parameter of 0.55nm. The local horizontal lattice spacing *Lx* of the RP fault (position number 9) is in fact the distance of (200) plane of the BaO (0.275nm), which matches well with our result. Such large lattice spacing would inevitably lead compression strains around the RP fault (position number 8 and 10). This in principle would also promote the intersection of TDs and RP faults for strain accommodations, as seen in Figs 3 and 4.

## Discussion

The RP faults, similar to RP phases, are normally rationalized as (AO)(ABO_3_)_n_ series of oxides, where for the RP faults the n is infinite. It has been demonstrated that the RP fault related structures show many exciting properties including superconducting^30^, unusual ferromagnetic behaviors^31^, and tunable dielectric property^32^. An important aspect is the introduction of the rock salt (AO) layers, that enables the control of the interactions between (ABO_3_)_n_ building blocks. Thus mapping the element distribution at an atomic scale and correspondent stoichiometry can provide further information on the role of rock salt layer and furthermore the influence on the properties of the RP fault related structures. On the other hand, considering the density as well as the strains of the RP faults, a large amount of strains can be developed in the film. Such kind of strain at nano-scale would severely influence the physical behavior of thin films^33^,^34^. In addition, the chemistry character of these RP faults may also play a role in the performance of BSO thin films, as the local excess of Ba-O layer of the RP faults can not only act as a refraction center for carriers, but also lead to a fluctuation of compositions of thin films, causing the deviation of optimized carrier concentration.

It should be also emphasized that the generation mechanisms for the RP fault and the SF of 1/2a < 110 > are different. As previously mentioned above, the formation of the SF of 1/2a < 110 > is attributed to the dissociation of perfect dislocation, which only contains 5 ~ 7 layers of {110} plane for all our experiments. Such kind of SF have also been observed in other materials^27^. For the RP fault, it is a kind of as-grown defect and many aspects can influence its generation, such as the growth mode, mismatch with the substrate and the stoichiometry of target^25^,^35^. Films growth in island mode can often contain planar defects at interface of islands^25^, whereas those using target with A site element excess have also been confirmed to generate RP fault^35^.

## Methods

A 0.001% La-doped BSO thin film was grown on a (LaScO_3_) LSO-buffered (SrTiO_3_) STO (001) substrate by pulsed laser deposition (PLD) technique. The 1 inch-diameter LSO and 0.001% La-doped BSO targets were synthesized using conventional solid state method starting from stoichiometric La_2_O_3_, BaCO_3_, SnO_2_ and Sc_2_O_3_ powers. PLD experiment was performed using a 248 nm KrF excimer laser with laser frequency 4 Hz. Vacuum chamber was pumped to 10^−7^ Torr and then filled with 1 mTorr O_2_. Substrate temperature was kept at 850 °C during film deposition, and a LSO buffer layer was first grown on a STO (001) substrate with laser energy density 3.85 J/cm^2^ for 5 minutes. BSO film was then grown on the LSO buffer layer with 2.1 J/cm^2^ for 30 minutes.

TEM samples for both cross-sectional and plan-view analysis were prepared by conventional procedure, including slicing, grinding, and final ion milling, while the plan-view samples were milled only from the substrate side. A Tecnai G^2^ F30 transmission electron microscope was used for electron diffraction, and diffraction contrast analysis. A Titan G^2^ 60–300 microscope with a high-brightness field-emission gun, double aberration (Cs) correctors and a Gatan GIF was used to acquire HAADF-STEM images and EELS. To quantitatively analysis the structural variation according to atomic resolved HAADF-STEM images, positions of atoms were then determined by fitting the intensity profiles of every atom column based on two-dimensional Gaussian fitting using Matlab^29^.

## Additional Information

**How to cite this article**: Wang, W. Y. *et al.* Atomic mapping of Ruddlesden-Popper faults in transparent conducting BaSnO_3_-based thin films. *Sci. Rep.* **5**, 16097; doi: 10.1038/srep16097 (2015).

## Supplementary Material

Supplementary Information
