# Supplementary material for: Atomic mapping of Ruddlesden-Popper faults in transparent conducting BaSnO3-based thin films
Source: Sci Rep. 2015 Nov 3;5:16097. doi: 10.1038/srep16097 (PMC4630648; doi:10.1038/srep16097)
Supplement: Supplementary Information [file srep16097-s1.pdf]

Supplementary Materials

Atomic mapping of Ruddlesden-Popper faults in transparent conducting $BaSnO_3$-based thin films

W. Y. Wang[1], Y. L. Tang[1], Y. L. Zhu[1]*, J. Suriyaprakash[1], Y. B. Xu[1], Y. Liu[1], B. Gao[2], S-W. Cheong[2], X. L. Ma[1]*

[1]Shenyang National Laboratory for Materials Science, Institute of Metal Research, Chinese Academy of Sciences, Wenhua Road 72, Shenyang 110016, China.
[2]Rutgers Center for Emergent Materials and Department of Physics & Astronomy, Rutgers University, Piscataway, New Jersey 08854, USA.

Correspondence should be addressed to Y. L. Zhu(ylzhu@imr.ac.cn) or X. L. Ma (xlma@imr.ac.cn).

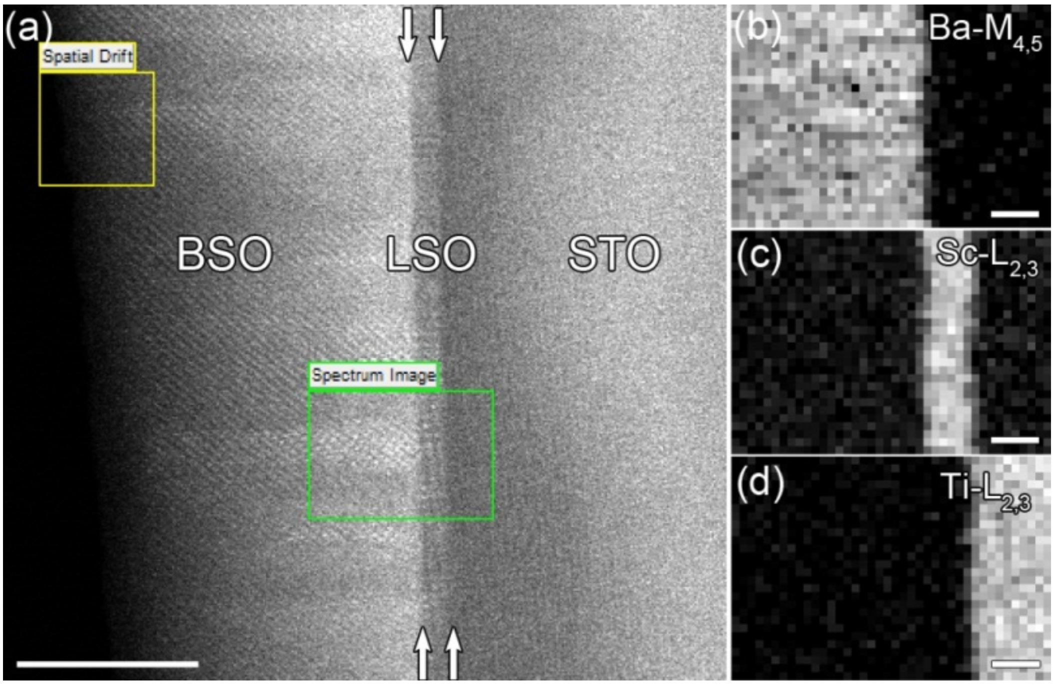

Figure S1. Element mappings of BSO/LSO/STO thin film. (a) A low magnification HAADF-STEM image of the BSO/LSO/STO thin film. The interfaces are indicated by arrows. The scale bar is 50 nm. (b-d) EELS element mappings of the area marked by the red frame in (a). The scale bar is 10 nm. Note that the bilayer structure is clearly shown by the element mappings.

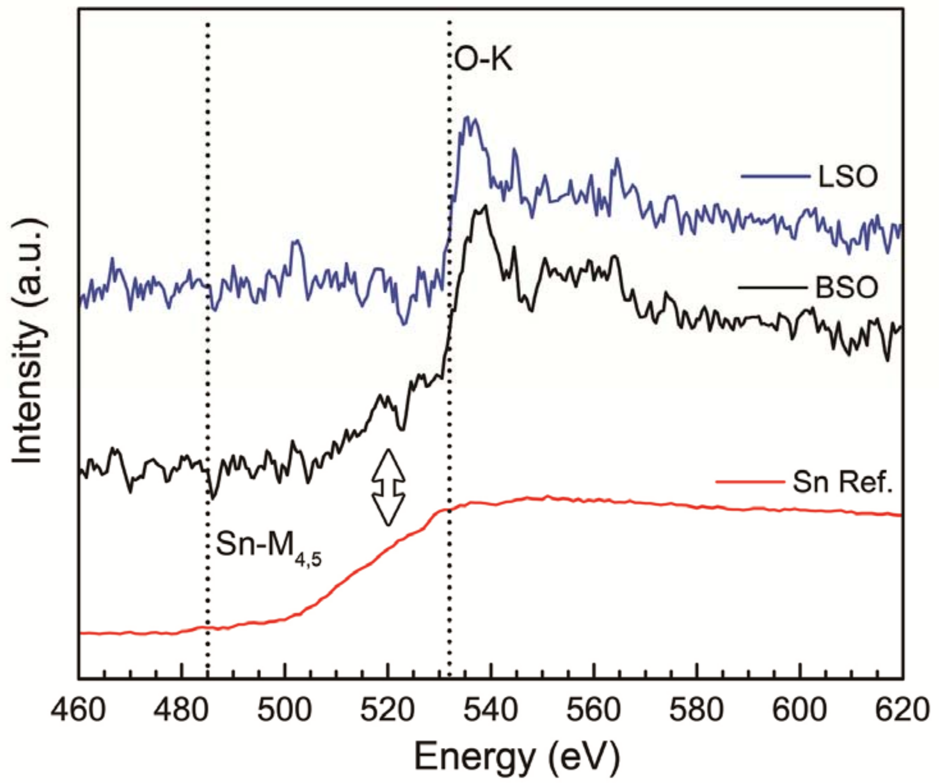

Figure S2. Electron energy loss spectrums of Sn and O. The spectrum of BSO is in black line and that of LSO ($LaScO_3$) is in blue. A reference spectrum of Sn (red line) is used based on the EELS Atlas of Gatan DigitalMicrograph. The thresholds of Sn-$M_{4,5}$ (485 eV) and O-K (532 eV) are marked by vertical dotted lines. The delayed feature of Sn-$M_{4,5}$ is obvious and is indicated by the arrow. Note that the difference between Sn-M and O-K is apparent, although they are partly mixed together in BSO.

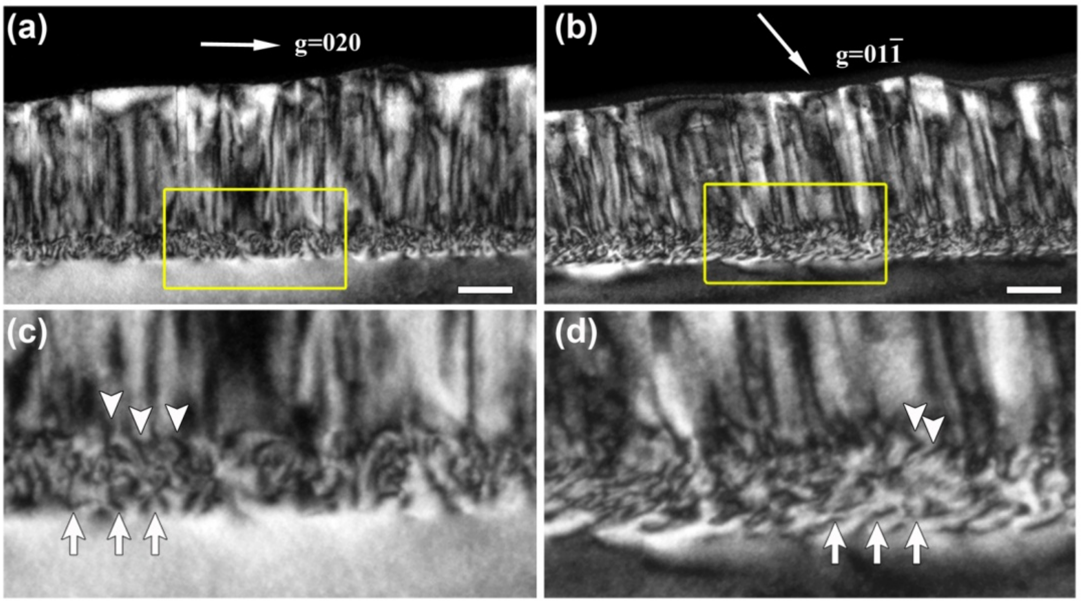

Figure S3. Contrasts of misfit dislocations at BSO/LSO and LSO/STO interfaces. (a)-(b) Two-beam dark field images of BSO/LSO/STO thin film using (020) and (01-1) reflections of [100] pole, which are corresponding to Figure 2(b) and Figure 2(c). The scale bar is 50 nm. (c)-(d) The enlargement of the areas marked by the rectangular frames in (a) and (b) showing the details of contrasts at the interfaces. The short dark lines existing at the interfaces are marked by two types of arrows, respectively. Note that in Figure S3(d) the short dark lines are lying at the interfaces which are commonly observed for misfit dislocations when obtained far away from the zone axis (Ref. S1 and Ref. S2). The structural features of these misfit dislocations are also demonstrated in Figure S4.

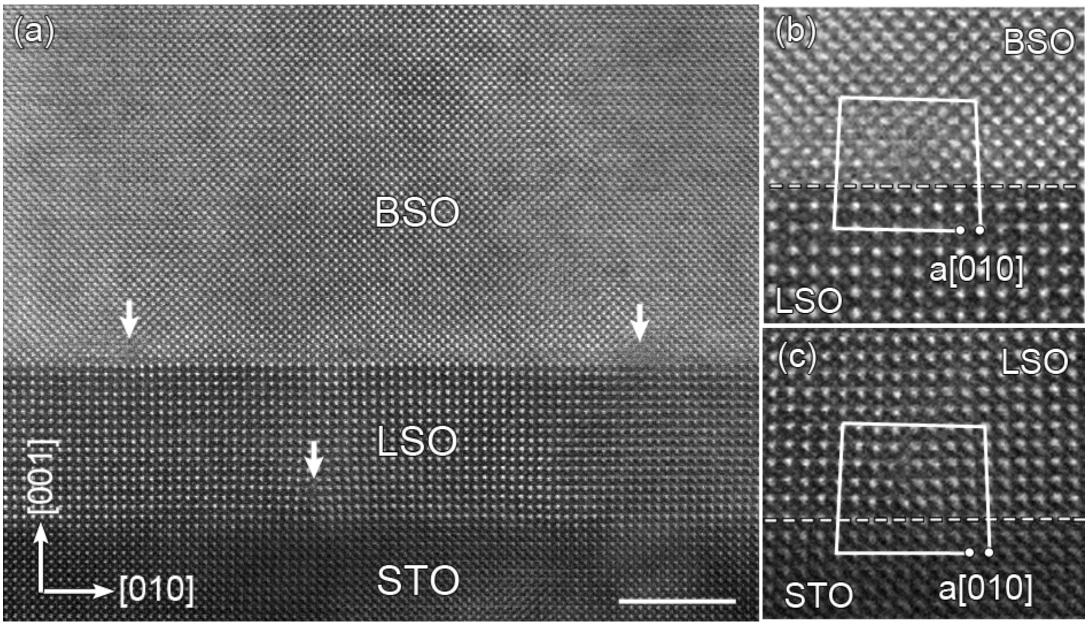

Figure S4. Characteristics of misfit dislocations of BSO/LSO/STO thin film. (a) A high magnification HAADF-STEM image of BSO/LSO/STO thin film in [100] direction. The misfit dislocations near interfaces of BSO/LSO and LSO/STO are marked by arrows. The scale bar is 5 nm. (b) An enlarged HAADF-STEM image of the misfit dislocation near BSO/LSO interface (left one in (a)). The Burgers vector is a[010]. The interface is marked by dotted line. (c) An enlarged HAADF-STEM image of the misfit dislocation near LSO/STO interface. The Burgers vector is also a[010]. The interface is marked by dotted line.

Reference:

[S1] Oh, S. H. Misfit strain relaxation by dislocations in $SrRuO_3$/$SrTiO_3$ (001) heteroepitaxy. *J. Appl. Phys.* **95**, 4691-4704, (2004).

[S2] Tang, Y. L., Zhu, Y. L., Meng, H., Zhang, Y. Q. & Ma, X. L. Misfit dislocations of anisotropic magnetoresistant $Nd_{0.45}Sr_{0.55}MnO_3$ thin films grown on $SrTiO_3$ (110) substrates. *Acta Mater.* **60**, 5975-5983, (2012).
